# Management of Chronic Hyperkalemia in Patients With Chronic Kidney Disease: An Old Problem With News Options

**DOI:** 10.3389/fmed.2021.653634

**Published:** 2021-06-04

**Authors:** Enrique Morales, Paolo Cravedi, Joaquin Manrique

**Affiliations:** ^1^Department of Nephrology, Hospital Universitario 12 de Octubre, Madrid, Spain; ^2^Instituto de Investigación Hospital Universitario 12 de Octubre (imas12), Madrid, Spain; ^3^Department of Medicine, Universidad Complutense de Madrid, Madrid, Spain; ^4^Department of Medicine, Icahn School of Medicine at Mount Sinai, New York, NY, United States; ^5^Nephrology Department, Complejo Hospitalario de Navarra, Pamplona, Spain; ^6^Navarra Institute for Health Research, IdiSNA, Pamplona, Spain

**Keywords:** hyperkalemia, chronic kidney disease, RAASi, potassium binders, patiromer, zirconium

## Abstract

Hyperkalemia is one of the main electrolyte disorders in patients with chronic kidney disease (CKD). The prevalence of hyperkalemia increases as the Glomerular Filtration Rate (GFR) declines. Although chronic hyperkalemia is not a medical emergency, it can have negative consequences for the adequate cardio-renal management in the medium and long term. Hyperkalemia is common in patients on renin-angiotensin-aldosterone system inhibitors (RAASi) or Mineralocorticoid Receptor Antagonists (MRAs) and can affect treatment optimization for hypertension, diabetes mellitus, heart failure (HF), and CKD. Mortality rates are higher with suboptimal dosing among patients with CKD, diabetes or HF compared with full RAASi dosing, and are the highest among patients who discontinue RAASis. The treatment of chronic hyperkalemia is still challenging. Therefore, in the real world, discontinuation or reduction of RAASi therapy may lead to adverse cardiorenal outcomes, and current guidelines differ with regard to recommendations on RAASi therapy to enhance cardio and reno-protective effects. Treatment options for hyperkalemia have not changed much since the introduction of the cation exchange resin over 50 years ago. Nowadays, two new potassium binders, Patiromer Sorbitex Calcium, and Sodium Zirconium Cyclosilicate (SZC) already approved by FDA and by the European Medicines Agency, have demonstrated their clinical efficacy in reducing serum potassium with a good safety profile. The use of the newer potassium binders may allow continuing and optimizing RAASi therapy in patients with hyperkalemia keeping the cardio-renal protective effect in patients with CKD and cardiovascular disease. However, further research is needed to address some questions related to potassium disorders (definition of chronic hyperkalemia, monitoring strategies, prediction score for hyperkalemia or length for treatment).

## Introduction

Potassium regulates many biological processes and plays a main role in human physiology. Among the total body electrolyte composition, 2% potassium resides in extracellular compartment and 98% in the intracellular space. Vital physiological processes are based on the balance of potassium regulation, including acid-base homeostasis, systemic blood pressure control and vascular tone, hormone secretion, carbohydrate metabolism, gastrointestinal motility, fluid and electrolyte equilibrium, the resting cellular-membrane potential and action potentials in neuronal, muscular, and cardiac tissue (1, 2). An average adult has approximate 50 mmol of potassium per kg, meaning 3,500 mmol of total body potassium. The normal kidney maintains normal potassium homeostasis regardless of the total amount of dietary intake. Approximately 90% of the daily potassium intake is excreted in the urine; the rest is excreted by the gastrointestinal tract, leading a <10% of variations in the plasma potassium level during the course of a day. Fresh fruits and vegetables, are considered healthy choices for most people and they are associated with a less renal complications except for hyperkalemia (3). Nevertheless, among patients with chronic kidney disease (CKD), a higher dietary potassium intake may be associated with a higher risk of kidney disease progression (4).

Potassium disorders are common in patients with CKD, due to low glomerular filtration rate (GFR) and tubular disorders and represent a potentially fatal condition. Hyperkalemia represents a frequent complication of CKD progression, limiting the therapeutic strategies options recommended for treatment and prevention of cardiovascular disease (CVD), including renin-angiotensin-aldosterone inhibitors (RAASi) (5). An important CKD-related condition that contributes to hyperkalemia is metabolic acidosis, which causes a shift of potassium from the intracellular to the extracellular space (6). An unmet need exists for hyperkalemia management in the daily setting of special populations as CKD patients. The use of potassium binders has been controversial due to tolerability and safety profile, therefore new released, Patiromer and Zirconium, rather than the classic Sodium Polystyrene Sulfonate (SPS) could represent a strategy for the optimal manage of potassium disorders.

## Classic Feedback Regulation and Feedforward Control: Two Mechanisms of Potassium Regulation

Dietary potassium intake initiates both increased potassium excretion and sequestration in liver and skeletal muscle, an effect drived by insulin, catecholamines, alkalosis, and mineralocorticoids. Postprandial insulin not only regulates the serum glucose concentrations but also shifts dietary potassium into cells until the kidney starts potassium excretion. Kaliuresis is driven by two mechanisms, depending on the plasma potassium level (the classic feedback regulation) or independent of the plasma potassium level (feedforward regulation) (5). The healthy kidney has the capacity to excrete high amounts of potassium. In the absence of CKD, humans can intake very large amounts without developing clinically significant hyperkalemia. If the quantity of released potassium is sufficient to increase the plasma potassium level, the feedback system is activated. The potassium is freely filtered by the glomerulus and almost completely reabsorbed in the tubule, just a small portion reaches the distal nephron. A high potassium dietary intake is related to a inhibitory effect on sodium reabsorption in the proximal tubule and the thick ascending limb, facilitating increased delivery of Na^+^ to the aldosterone-sensitive distal nephron (ASDN), running a potassium fine regulation (1). A potassium sensor in the distal tubule has also been described as complementary mechanism in the potassium regulation.

Two mechanisms have been proposed as the major drivers for the proximal tubule reabsorption of 60–75% of filtered potassium are solvent drag and electrodiffusion. Several co-transporters and ion channels are involved in the complex regulatory reabsorption system of the 15–20% of filtered potassium in the thick ascending limb of the loop of Henle. The best characterized is the sodium potassium chloride cotransporter also known as NKCC2 (7). NKCC2 has one of the highest overall reabsorptive capacities in the kidney. The distal convoluted tubule mediates reabsorption of 5–10% of filtered potassium. In the early segment of the distal convoluted tubule, potassium transport is driven exclusively by the thiazide-sensitive sodium chloride cotransporter, whereas in the later segment of the ASDN, the epithelial sodium channel also participates. Three main factors regulate potassium secretion in the ASDN: (1) sodium load, (2) fluid flow rate, and (3) aldosterone and catecholamines (7).

**Figure 1:**
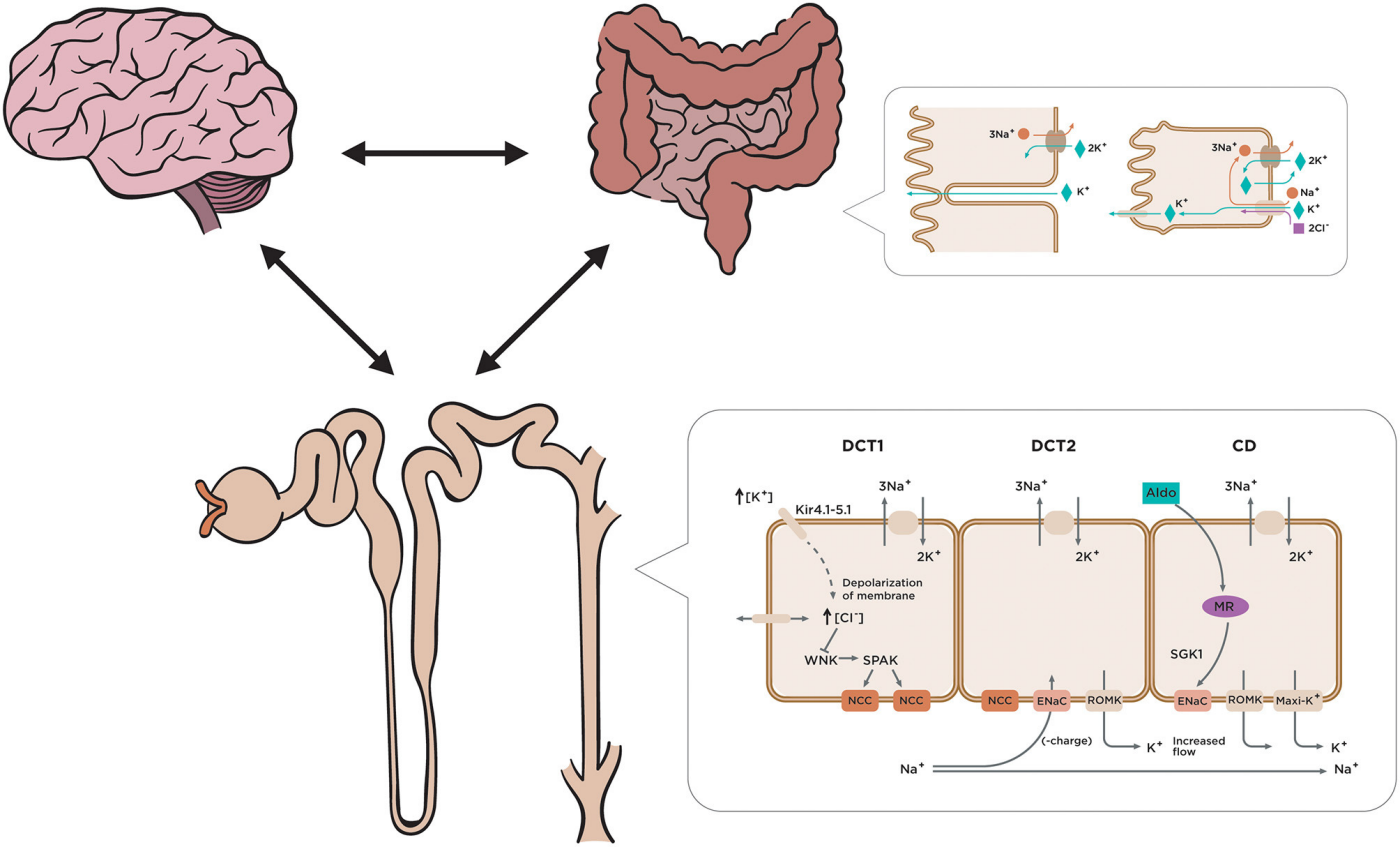
Regulation of external and internal potassium balance. So-called feed-forward control refers to a potassium control pathway that responds to a pre-determined signal from the organism and is highly relevant to the mechanism of additional potassium regulation. The brain generates a regulatory circuit with the kidney and the colon that anticipates the presentation of food. Gastrointestinal-renal signals with a kaliuretic effect are generated, which will be able to mediate renal potassium elimination independent of changes in serum potassium and aldosterone concentration. Several co-transporters and ion channels are involved in the complex regulatory system of potassium reabsorption. The distal convoluted tubule mediates reabsorption of 5–10% of filtered potassium. Increased plasma K^+^ concentration depolarizes cells in the proximal portion of the distal convoluted tubule (DCT1) through effects dependent on the potassium. Increased Na^+^ delivery and flow to the downstream distal portion of the DCT where aldosterone sensitivity begins (DCT2, connecting tubule, and collecting duct) along with increased aldosterone levels drive potassium secretion.

**Figure 2:**
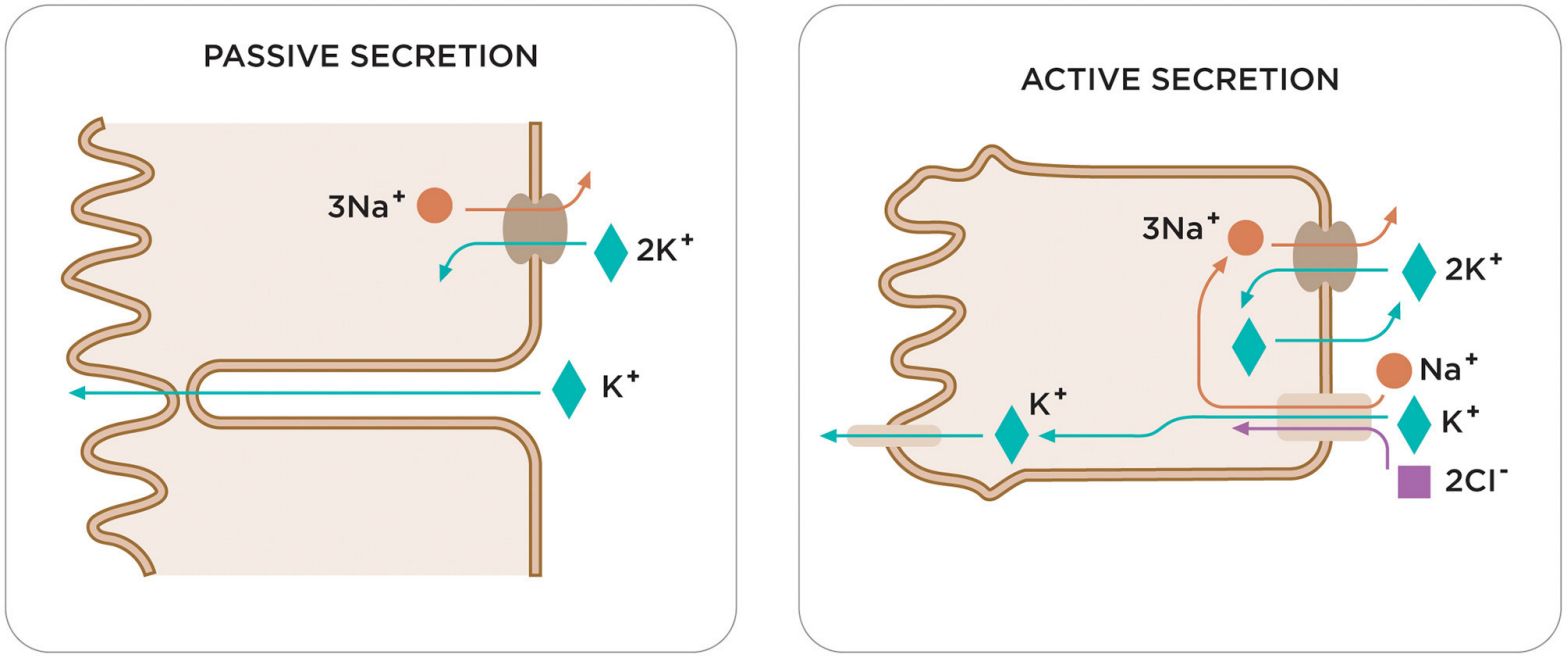
Two compensatory mechanisms in the colon respond to elevated serum potassium levels in patients with CKD. Current studies highlight the existence of a feed-forward control in the regulation of potassium homeostasis, capable of causing rapid changes in renal potassium excretion. Among the different elements of this feed-forward control, the colon plays a fundamental role in the regulation of potassium. It is worth noting the different transport capacity of the potassium in the various segments of the colon or the different expression or activity of potassium channels on the membrane apical of the colon (BK channels). Although the role of the colon in excretion of potassium is not well-known yet, recent studies have found that in CKD the colon is responsible for a considerable increase in potassium removal, which is attributed to an increase in activity of the BK channels. There are two compensatory mechanisms in the colon in response to elevated serum potassium levels in patients with CKD: passive and active secretion.

Potassium homeostasis is regulated by renal and extrarenal mechanisms. To enhance the ability to eliminate potassium, there is a complementary mechanism called the “feedforward control.” As the GFR declines, potassium excretion is compensated in the remnant functional kidney by enhancing its ability excreting this excess of potassium (1). It is also well-described that hyperkalemia does not appear until GFR reaches the threshold of 15 ml/min, earlier if aldosterone dysfunction is associated. Beyond this adaptive response, extra-renal contribution to potassium handling, especially colonic excretion, becomes critical to prevent acute or chronic hyperkalemia and makes a substantial contribution to potassium homeostasis in patients with CKD (8). Potassium homeostasis in the bowel is modulated due to an enteric sensing system (gut factor), that enhances kaliuresis once potassium enters the intestine, the feedforward system initiated at splanchnic receptors provides maintenance of total body potassium levels within narrow limits after the ingestion of a meal (2, 5) (Figure 1). There are two compensatory mechanisms in the colon in response to elevated serum potassium levels in patients with CKD: passive secretion which is responsible for net colonic potassium secretion, basically in the distal colon, and active secretion, that occurs throughout the colon and mechanistically follows the “pump leak” model (basolateral uptake of potassium via Na/K pump, Na/K/Cl cotransporter and efflux of potassium through BK channels) (9) (Figure 2). Conversely, during fasting, potassium is released from intracellular stores (liver, muscles). Both feedback and feedforward control work together to maintain potassium and sodium homoeostasis (10). Daily potassium intake, renal management and enteric sensor are not the only players in the potassium homeostasis. There is a central circadian clock that regulates the nightly and daily kaliuresis. This rhythm synchronizes the renal tubule cells with the brain, and increases excretion during the active daylight phase and diminishes it during the inactive nighttime phase, regardless from activity, posture or dietary intake (2).

Disorders of potassium balance are common due to changes in dietary intake, GFR, or physiological management (renal and gastrointestinal) among different clinical settings, as CKD patients, renal transplantation (RT), resistant hypertension, or cardiorenal syndrome. The use of otherwise recommended medication like RAASi or MRAs could be critical in the incidence and severity of hyperkalemia.

Traditional dietary recommendations to CKD patients limit the intake of fruits and vegetables because of their high potassium content. However, there is a controversy based on to the benefits derived from a fundamentally vegetarian diet (3). Western diets are largely acid-producing since they are deficient in fruits and vegetables and rich in animal proteins that can induce metabolic acidosis in CKD patients. Thus, diets rich in vegetables and fruits might lower the dietary acid load and induce similar beneficial results as an alkali therapy in CKD patients (11, 12).

## Chronic Hyperkalemia in Patients With Kidney Disease

### Hyperkalemia in Patients With CKD Non-dialysis

Studies in patients with CKD have shown a remarkable frequency in hyperkalemia in advanced CKD stages, hyporreninemic hypoaldosteronic diabetic patients, renal transplantation (RT), and patients with RAAS inhibition (4, 6). Available information shows prevalence percentages ranging from 5 to 20% depending on the stage of CKD (13). The burden of hyperkalemia is remarkable not only in terms of prevalence, but also in terms of patient survival even for serum potassium levels only moderately increased (14, 15). Compensatory mechanisms may help improving tolerance to hyperkalemia in patients with CKD and a J-shaped correlation was found between serum potassium and overall mortality risk in non-dialysis patients (16). Moreover, new onset or persistence of mild-to-moderate hyperkalemia (potassium 5.0–6.0 mmol/L) during 12 months of observation significantly increased by 30% the risk of End Stage Renal Disease (ESRD) (4, 17, 18). It has been suggested that CKD patients adapt to elevated potassium concentrations through modifications in gastrointestinal secretions which may favor intracellular potassium storage, or by increasing insulin-mediated intracellular potassium uptake in splanchnic and peripheral muscle tissues (19). On the other hand, relevant are also the related economic costs that double in the presence of persistent hyperkalemia (20). In a recent retrospective cohort study of 1,499 patients with chronic hyperkalemia and CKD, heart failure or diabetes mellitus followed up for 36 months, the annual healthcare cost per patient with severe hyperkalemia has calculated as double compared to mild hyperkalemia (21).

### Hyperkalemia in Patients With Renal Transplantation

Hyperkalemia is a common complication in RT with a reported incidence ranging from 25 to 44% in RT on calcineurin inhibitors (CNIs) (22). It is a life-threatening complication that may increase the length of stay (23). Hyperkalemia in the RT is usually seen in association with renal tubular acidosis and can occur even without insulin deficiency, metabolic acidosis, decreased eGFR or decreased distal sodium delivery. Insulinopenia or insulin resistance can disturb the shifting of potassium and glucose from the extracellular to the intracellular compartment and developing hyperkalemia in the post-transplant setting, especially in insulin dependent diabetics (22). Also the medications used post-transplant has been described as a major cause for post-transplant hyperkalemia in RT, even in those with a well-working graft. As mentioned above, CNIs are considered the major players in the development of hyperkalemia in the RT (24). The use of trimethoprim in standard doses can contribute to hyperkalemia by ENaC blockade. The use of RAASi is recommended after transplantation associated to a better patient and graft survival in RT, but the risk of life-threatening hyperkalemia is twice as the risk for recipients not on these medications (25). Table 1 shows the most recent options of treatment of hyperkalemia in RT, highlighting the scarce number of patients included in various studies, especially with the new potassium binders.

**Table 1:** Clinical studies of Patiromer and SZC in transplant patients.

**Study**	**Drug**	**Population**	**Primary outcomes**
Schnelle et al. (26)	Patiromer 8.4 g daily	SOT: kidney 73%, liver 21%, kidney-pancreas 3%, lung 3% *N* = 37	Moderate reduction in Potassium levels at week 4 and 12. Increase of TC levels.
Lim et al. (27)	Patiromer 8.4–16.8 g daily	Kidney transplants *N* = 17	K <5.2 mmol/l at last follow-up (84%). Seven patients required TC dose reduction.
Rattanavich et al. (28)	Patiromer 8.4–16.8 g daily	2 kidney transplants	Patiromer is effective and does not affect TC levels.
Winstead et al. (29)	SZC	SOT: kidney 45.7%, liver 40%, heart 5.7%, kidney-liver 5.7%, kidney-heart 2.9% *N* = 35	Potassium levels decreased by −1.3 mmol/l from day 0 to day 7. TC −0.54 ng/ml.

*SOT, Solid Organ Transplantation; SZC, Sodium Zirconium Cyclosilicate; TC, Tacrolimus*.

### Hyperkalemia in Patients With Resistant Hypertension

Resistant hypertension (RHT) is defined by the failure of the recommended treatment strategies (at least three drugs, including a diuretic) to reduce systolic (SBP) and diastolic blood pressure (DBP) values to <140 mmHg and/or <90 mmHg (30). The PATHWAY-2 randomized trial suggested that RHT is commonly a salt-retaining state, most likely due to inappropriate aldosterone secretion (31). In accordance with these findings, in recent years, an increasing body of evidence has shown a beneficial effect of MRAs, such as eplerenone and spironolactone, in improving BP control in patients with RHT. A recent meta-analysis based on data from multiple RCTs provides the evidence that add-on use of spironolactone in patients with RHT is effective in lowering SBP and DBP (32). Nevertheless, the hyperkalemic effect of MRAs in the treatment of hypertension limits their use in patients with advanced CKD. The latest guidelines from the European Society of Hypertension and the American Heart Association established that, besides optimal doses or best tolerated doses of an optimal therapy, the fourth-line treatment should involve a blockade of aldosterone through the use of MRAs (30, 33). However, it is suggested that the use of spironolactone must be restricted to patients with an eGFR > 45 mL/min and a potassium <4.5 mmol/L. For these reasons, it is important to provide the nephrologist with tools to control potassium while implementing the cardiology guidelines in patients with CKD and RHT. Recently, the AMBER study has suggested that Patiromer enables the use of spironolactone, which effectively lowers systolic blood pressure in patients with RHT and CKD. Persistent spironolactone enablement in this population of patients has clinical relevance for the treatment of RHT (34).

## What Are the Consequences of Decreasing or Discontinuing RAAS Inhibitors?

The renoprotective effects of RAASi should be balanced against the associated risk of hyperkalemia, especially in patients with CKD. Although these drugs have shown an important benefit in patients with CKD, diabetic renal disease, HF and reduced ejection fraction (HFrEF) (35) and RT, hyperkalemia limits its use contributing to treatment withdrawal or underprescription (6, 36).

Current nephrology guidelines recommend RAASi as the primary therapeutic tool in patients with urine albumin excretion >300 mg/24 h or proteinuria >500 mg/24 h based on the proven renoprotective efficacy of these agents in proteinuric patients (37). RAASi enable preserving kidney function and delay the progression to ESRD in CKD (37, 38). Nevertheless, side effects limit their use, particularly associated to diuretics (39), dual blockade of RAAS [“Ongoing Telmisartan Alone and in combination with Ramipril Global Endpoint Trial,” ONTARGET (40), or “Aliskiren Trial in Type 2 Diabetes Using Cardiorenal Endpoints,” ALTITUDE] (41, 42), or neprylisin inhibitors (43). Hyperkalemia is more common than acute kidney injury (AKI) with an incidence ranging from 5 to 40%, and, moreover, patients who would otherwise benefit from RAASi either do not receive these medications, receive suboptimal doses, or discontinue therapy (44). An observational retrospective Japanese cohort study showed that 54% of patients discontinued RAASi after hyperkalemia (45). Controversy came up with the VA-NEPHRON-D trial that showed a strong trend to lowering the risk of renal disease progression with dual RAAS blockade vs. monotherapy (40). In the same way, MRA therapy could offer an additive proteinuria lowering effect but, like the dual blockage, increases the risk of hyperkalemia (46). Mortality rates are higher with suboptimal dosing/discontinuation of RAASi among patients with CKD, diabetes or HF compared with those with full RAASi dose (19, 47).

**Figure 3:**
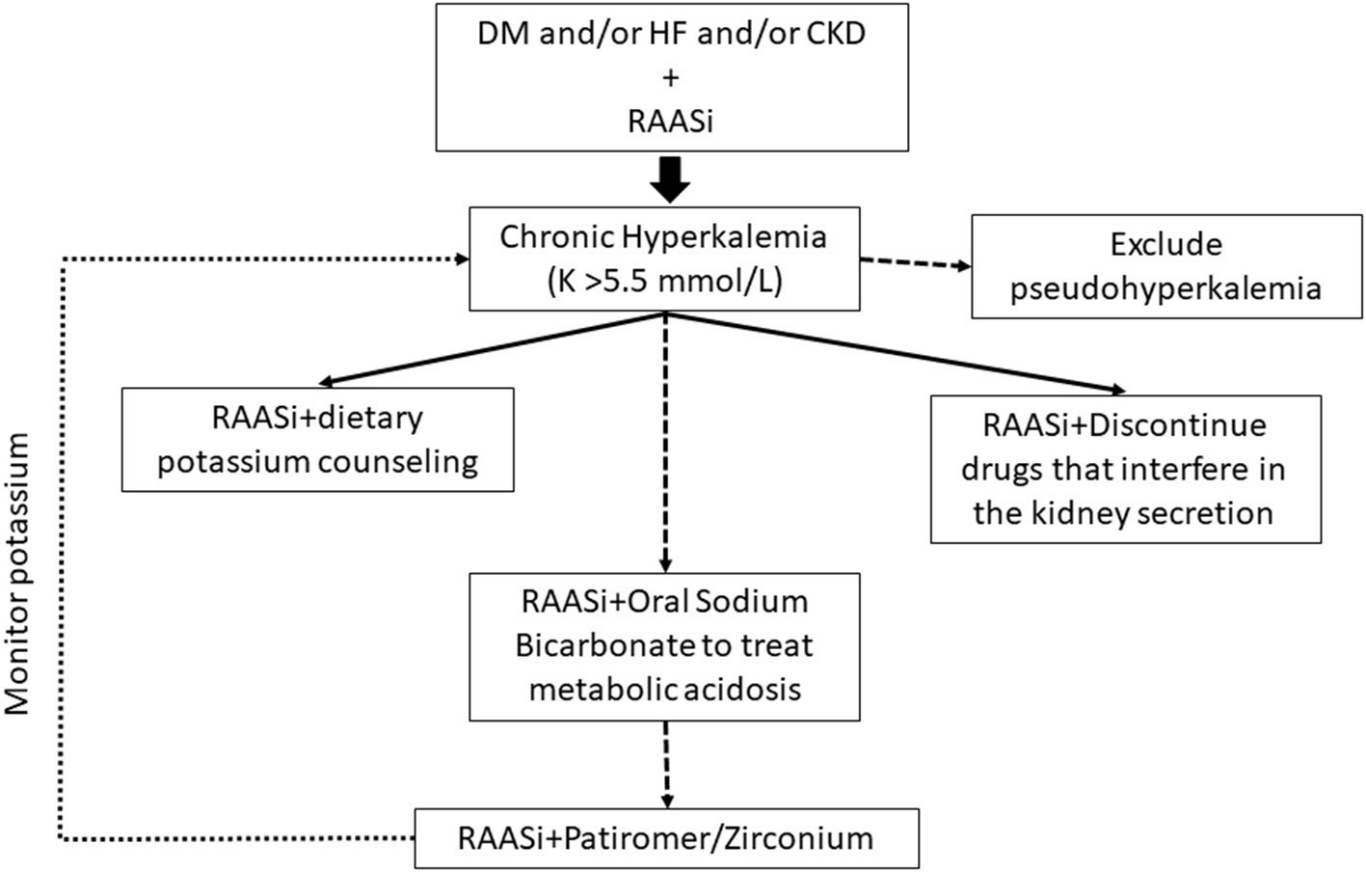
Treatment algorithm for chronic hyperkalemia. Current recommendations regarding the management of chronic hyperkalemia (long-term elevated serum potassium) include the management of diuretics, modification of RAASi dose, treatment of metabolic acidosis with sodium bicarbonate, and removal of other hyperkalemia-causing medications. A team approach for chronic hyperkalemia, primary care physicians, nurses, pharmacists, or dietitians is optimal. The initiation of potassium binding agents should be considered in patients with chronic hyperkalemia despite optimized diuretic therapy and correction of metabolic acidosis. CKD, chronic kidney disease; DM, Diabetes Mellitus; HF, hearth failure; K, potassium; RAASi, renin-angiotensin-aldosterone system inhibitors.

In routine clinical practice, we faced the conundrum of prescribing RAASi assuming the likelihood of hyperkalemia (up to 54%) or avoiding/discontinuing beneficial RAASi therapies. Although hyperkalemia has long been regarded as a reason for RAASi non-prescription, down titration, or discontinuation, it has been disregarded as a major topic in the literature (47). Nevertheless, the gap in information on the incidence of hyperkalemia between the real world and the world of clinical trials is well-known. A recent Portuguese study in patients with HFrEF highlighted their higher risk of developing hyperkalemia associated to comorbidities such as CKD, diabetes, or the use of dual or triple therapy including RAASi and concurrent HF therapy. In the same way the initiation of RAASi compared with calcium channel blockers may confer kidney and cardiovascular benefits among patients with advanced CKD from the Swedish Renal Registry (48). This point emphasizes the need to carry out studies on the impact of the reduction or suspension of RAASi on the prognosis of these patients (41). This significant body of literature highlights the need for a change of therapeutic strategy in maintaining the renal and cardio protection of RAASi in selected population as CKD patients, HF patients or diabetic patients, and the new potassium binders become essential treating hyperkalemia (Figure 3).

## Treatment of Chronic Hyperkalemia: Something Old

In a recent meta-analysis of 1,217,986 participants in 27 diverse cohorts, hyperkalemia (serum K ≥ 5.0 mmol/L) was associated with significantly higher long-term risk of all-cause and CV mortality, and of ESRD. Although the definition of hyperkalemia is debated, ideal outcomes were observed with serum potassium concentrations of 4–4.5 mmol/L (15). Chronic management of hyperkalemia usually starts by dietary education and recommendation of a low potassium intake. However, it is well-known the renoprotective effect of potassium supplementation or intake by fruit and vegetable in CKD (49). It is well-known the renoprotective effect of potassium supplementation or intake by fruit and vegetable in CKD. Although the acknowledgment that dietary potassium restriction is a valid strategy to treat acute hyperkalemia, it's been hypothesized that potassium restriction as a general strategy to prevent hyperkalemia in persons with CKD may deprive patients of the beneficial effects associated with potassium-rich diets (9). The “KDIGO 2020 Executive Conclusions on Potassium Hemostasis and Disorders,” did not find evidence that increased potassium intake, or liberalization of potassium restrictions, in patients with advanced CKD would be safer (9). The position statement of the Italian Society of Nephrology for Hyperkalemia management in CKD patients state that potassium ≥5.0 mmol/L must be considered pathologic in CKD and require careful follow-up and implementation of preventive and therapeutic strategies aimed at maintaining it in the optimal clinical range (50). On this regard, it is important to highlight the role of new potassium binders to overcome potassium restrictions in CKD (51, 52).

In healthy subjects the gastrointestinal tract contribution to potassium excretion is minimal (about 10% of the total), while in the case of kidney disease it may increase until it accounts for 50% of the total potassium excretion in patients on dialysis. The kidney (feedback control) and the colonic potassium handling (feedforward control) are strictly related, and it has been shown that the potassium enteral load may influence renal excretion (gut-dependent kaliuresis sensor, mentioned above) (53). This potassium secretory capacity makes of the colon a potential target for therapies aimed at treating and preventing hyperkalemia in patients with advanced CKD. Potassium binders make potassium unavailable in the distal colon for absorption by trapping it within the binder molecule, which is then excreted with the feces. Historically, the only options for promoting potassium elimination by the gastrointestinal tract have been limited to the “old” sodium cation-exchanging resin, sodium polystyrene sulfonate (SPS, Kayexalate; Sanofi-Aventis US LLC, Bridgewater, NJ). SPS was approved in 1958 but despite its common use, there is limited evidence demonstrating the effectiveness and safety in controlled studies (Table 2). The mixture with sorbitol at high concentrations, moreover, carries a risk of colonic necrosis and other serious GI adverse events (60, 61). A significant increase in the incidence of hospitalization for serious adverse GI events has been recently described in a large cohort of SPS users, when compared with matched non-users (62). Moreover, SPS initiation in adults with CKD stages has been associated with a higher incidence of severe gastrointestinal adverse events, mainly ulcers and perforations, possibly in a dose-dependent manner (63).

**Table 2:** Clinical studies of sodium and calcium polysterene sulfonate (54).

**Study**	**Drug**	**Population**	**Primary outcomes**
Phase 4, randomized, double-blind, placebo controlled; (55)	SPS 30 g or placebo QD	Pre-dialysis (stages 3–5), EGFR <40 ml/min and potassium 4.5–5.5 mmol/L *N* = 33	Mean change in serum Potassium was superior to placebo in reducing serum potassium over 7 days vs. placebo: −1.04 mmol/l (−1.37 to 0.71 mmo/L)
Effect of SPS in CKD; (56)	4 single-dose SPS and placebo on 5 different test days	Patients with CKD *N* = 6	No significant effect of SPS on total potassium output
Randomized and crossover design; (57)	CPS vs. SPS therapy for 4 weeks	Pre-dialysis CKD 4–5 and Potassium >5 mmol/L *N* = 20	CPS safer for the treatment of hyperkalemia in pre-dialysis patients, because it did not induce hyperparathyroidism or volume overload
Randomized Control trial; (58)	CPS vs. SPS therapy	CKD stages 1–4 and Potassium >5.2 mmol/L *N* = 97	Both CPS and SPS can be used effectively for reducing hyperkalemia of CKD. CPS showed fewer side effects as compared to SPS
Prospective, Randomized, Crossover Study; (59)	CPS 3-week × 5 g/day	HD patients and Potassium >5.5 mmol/L *N* = 58	CPS decreases serum levels of potassium and phosphorus in HD patients with interdialytic hyperkalemia. CPS does not induce volume overload or disrupt electrolyte balance.

*BB, Beta blockers; CKD, Chronic Kidney Disease; DM, Diabetes mellitus; CPS, Calcium polystyrene sulfonate; HD, Hemodialysis; HF, Heart Failure; HT, Hypertension; RAASi, Renin Angiotensin Aldosterone System Inhibitors; SPS, Sodium polystyrene sulfonate; QD, quaque die*.

In contrast, calcium polystyrene sulfonate (CPS) has long been used for patients with advanced CKD in some parts of the world. It avoids sodium retention and supplements calcium and may have an advantage over SPS. However, few clinical studies have evaluated the efficacy of CPS in the treatment of hyperkalemia (64) (Table 2).

## Treatment of Chronic Hyperkalemia: Something New

Two new colonic potassium binders have shown efficacy in lowering plasma potassium in recent clinical trials. Sodium Zirconium Silicate (SZC) and Patiromer have been introduced to manage hyperkalemia and promise to be more effective than SPS. In short and long-term studies involving patients on concomitant RAAS therapy, both SZC and Patiromer significantly lowered plasma potassium compared to placebo (Tables 3, 4). By facilitating fecal potassium excretion, these new binders are likely to open new horizon for the treatment and prevention of hyperkalemia in high-risk patients, such as those in therapy with RAASi and/or MRAs. These new potassium binders may allow extending these therapies to patients, in whom concerns with hyperkalemia have limited their use. Patiromer was approved for the treatment of hyperkalemia by the FDA in 2015. Patiromer is a cross-linked polymer of 2-fluoro acrylic acid (91%), with divinylbenzenes (8%) and 1,7-octadiene (1%). It is used in the form of its calcium salt (ratio 2:1) and with sorbitol, a combination called Patiromer sorbitex calcium. Patiromer works by binding the free potassium ions in the gastrointestinal tract, mainly in the distal colon lumen, and releasing calcium ions for exchange, thus lowering the amount of potassium available for absorption and increasing the amount that is excreted with the feces (41, 79).

**Table 3:** Clinical studies of patiromer (54).

**Study**	**Drug**	**Population**	**Primary outcomes**
PEARL-HF; phase 2, randomized, double-blind, placebo-controlled; (65)	Patiromer 15 g or placebo BID (+spironolactone 25 mg/d)^a^	CKD, HF, indication to initiate spironolactone, potassium of 4.3–5.1 mEq/L, receiving RAASi or BB *N* = 105	Mean change in serum Potassium: −0.22 mmol/l with Patiromer −0.23 mmol/l with placebo Mean difference vs. placebo: −0.45 mmol/L
OPAL-HK; phase 3,2 stages:(1) treatment, single-group, single-blind(2) withdrawal, randomized, single-blind, placebo controlled; (66)	Patiromer 4.2 g (mild hyperkalemia) or 8.4 g (moderate to severe hyperkalemia) BID	CKD (stage 3–4), eGFR 15 to <60 ml/min, receiving RAASi and serum potassium levels of 5.1 to <6.5 mmol/L *N* = 237	Treatment stage: Mean change in Potassium at week 4: Mild hyperkalemia −0.65 mmol/L Moderate to severe hyperkalemia −1.23 mmol/L Withdrawal stage: Median change in potassium week 4: 0 mmol/l with patiromer +0.72 mmol/l with placebo
AMETHYST-DN; phase 2, randomized, open-label; (67)	Mild hyperkalemia: Patiromer 4.2, 8.4, or 12.6 g BID^b^ Moderate hyperkalemia: patiromer 8.4, 12.6, or 16.8 g BID^b^	Type 2 DM and CKD (eGFR 15–60 ml/min) and serum potassium <5 mmol/l with RAASi *N* = 306	Mild hyperkalemia: Mean change in serum potassium: −0.35 mmol/l with Patiromer 4.2 g −0.51 mmol/l with Patiromer 8.4 g −0.55 mmol/l with Patiromer 12.6 g Moderate hyperkalemia: Mean change in serum potassium: −0.87 mmol/l with Patiromer 8.4 g −0.97 mmol/l with Patiromer 12.6 g −0.92 mmol/l with Patiromer 16.8 g
AMBER; phase 2, randomized, double-blind, placebo-controlled; (34)	Patiromer 8.4 g or placebo QD (+open-label spironolactone 25 mg/d)^c^	Uncontrolled resistant HT and CKD (eGFR 25–45 ml/min) and serum potassium 4.3–5.1 mmol/L *N* = 295	Patients remaining on spironolactone: 86% with Patiromer 66% with placebo More patients in Patiromer vs. placebo with serum potassium ≤ 5.5 mmol/L
DIAMOND; Phase 3 Patiromer for the Management of Hyperkalemia in Subjects Receiving RAASi for the Treatment of HF; (3)	Patiromer	Low ejection fraction heart failure (with or without CKD), receiving beta blocker, with either current hyperkalemia at screening or a history of hyperkalemia in the past year *N* = 2,400	Ongoing (NCT03888066)
PEARL-HD; Phase 4 Patiromer Efficacy to Reduce Hyperkalemia in ESRD; (68)	Patiromer	ESRD treated HD, two measured pre-dialysis K >5.5 mmol/l or one K >6.0 mmol/L *N* = 40	Ongoing (NCT03781089)
Single-center, randomized, open-label convenience sample pilot study in the ED; (69)	SOC or one dose of 25.2 g oral Patiromer plus SOC	Adult patients with ESRD and a serum potassium level of ≥6.0 mmol/L	2 h post treatment serum potassium with Patiromer was lower than SOC (5.90 vs. 6.51 mmol/L) and also 0.61 mmol/L lower than baseline
TOURMALINE: Open Label study, effect of Patiromer in hyperkalemic patients taking and not taking RAASi; (41)	Patiromer, 8.4 g/d to start, adjusted to achieve and maintain serum potassium of 3.8–5.0 mmol/L.	Hyperkalemia (potassium>5.0 mmol/L), receiving RAASi, BB or diuretics, CKD stages 1–5, HF and DM and/or HT *N* = 112	From baseline to week 4, the change in serum potassium was −0.67 mmol/l in patients taking RAASi and −0.56 mmol/l in patients not taking RAASi

*CKD, Chronic Kidney Disease; BB, Beta blockers; DM, Diabetes mellitus; ED, Emergency Department; HF, Heart Failure; ESRD, End-Stage Renal Disease; HT, Hypertension; RAASi, Renin Angiotensin Aldosterone System Inhibitors; SOC, Standard of care; quaque die; BID, Bis in die*.

a *Spironolactone dosage increased to 50 mg/d after 2 weeks in patients with serum K >3.5 to ≤ 5.1 mmol/L*.

b *Patiromer was titrated to achieve and maintain serum K ≤ 5.0 mmol/L*.

c *Spironolactone dosage increased to 50 mg/d after 2 weeks in patients with serum K >3.5 to ≤ 5.1 mmol/L*.

**Table 4:** Clinical studies of SZC (54).

**Study**	**Drug**	**Population**	**Primary outcomes**
Phase 2 study randomized, double-blind, placebo-controlled to assess safety and efficacy of SZC; (70)	0.3, 3, or 10 g of SZC three times daily for 2 Days or placebo	CKD (eGFR 30–60 ml/min) and moderate hyperkalemia (5–6 mmol/L), DM, HF, and HT *N* = 90	From baseline, mean serum potassium was significantly decreased by 0.92 ± 0.52 mmol/l at 38 h. Urinary potassium excretion significantly decreased with 10 g SZC as compared to placebo at Day 2
DIALIZE; phase 3b, randomized, double-blind, placebo-controlled; (71)	SZC 5, 10, or 15 g or placebo QD on non-dialysis days for 4 weeks.	HD 3 times, predialysis serum K>5.4 mmol/l (long interdialytic) and K>5 mmol/l (short interdialytic) *N* = 196	Maintenance of predialysis serum potassium 4.0–5.0 mmol/l during ≥ 3 of 4 hemodialysis sessions after long interdialytic interval without requiring rescue therapy: 41% with SZC 1% with placebo
ENERGIZE; phase 2, randomized, double-blind, placebo-controlled; (72)	SZC 10 g (3 doses in 10 h) or placebo	Emergency Department with potassium level >5.8 mmol/l *N* = 70	Mean change in serum Potassium at 4 h: −0.41 mmol/l with SZC −0.27 mmol/l with placebo Mean difference vs. placebo: −0.13 mmol/l
HARMONIZE; phase 3, 2-stage, randomized, double-blind, placebo controlled; (73)	Initial phase (open-label): SZC 10 g TID for 48 h Maintenance phase (double-blind): SZC 5, 10, or 15 g or placebo QD for 28 days	CKD <30 ml/min and hyperkalemia (potassium 5.1 mmol/L) *N* = 258	Initial phase (open-label): Mean change in serum potassium over 48 h: −1.1 mmol/l vs. baseline Maintenance phase (double-blind): 4.8 mmol/l with SZC 5 g QD 4.5 mmol/l with SZC 10 g QD 4.4 mmol/l with SZC 15 g QD 5.1 mmol/l with placebo vs. placebo for each dose
Phase 3 randomized, double-blind, placebo-controlled trial; (74)	Daily SZC (5, 10, or 15 g) or placebo for 28 days	HF patients with potassium ≥5.1 mmol/L *N* = 94	Compared with placebo, all three SZC doses lowered potassium and effectively maintained normokalemia for 28 days without adjusting RAASi
Phase 3 randomized, double-blind, two stages, placebo-controlled trial; (75)	SZC (1.25, 2.5, 5, or 10 g) or placebo three times daily for 48 h Initial phase and maintenance phase	CKD stage 3, serum potassium level 5–6.5 mmol/L *N* = 735	SZC showed a significant reduction in potassium levels at 48 h, with normokalemia maintained during 12 days of maintenance therapy as compared with placebo
Phase 2–3, randomized, double-blind, placebo-controlled, dose-response study; (76)	SZC 5, 10g, or placebo three times daily for 48 h	Japanese adults with hyperkalemia (Potassium>5.1 mmol/L) *N* = 103	At 48 h, the proportions of patients with normokalemia were 85.3, 91.7, and 15.2% with SZC 5 g, SZC 10 g, and placebo, respectively
ZS-005; phase 3, 2-stage, open-label; (77)	Correction phase: SZC 10 g TID for 24–72 h Maintenance phase: SZC 5 g QD	Hyperkalemia (two consecutive Potassium >5.1 mmol/L) *N* = 751	Correction phase: 78% of patients had serum Potassium 3.5–5.0 mmol/l at 72 h Maintenance phase: 88% of patients had serum Potassium <5.1 mmol/l over 3–12 mo
*Post-hoc* analysis of an open-label, single-arm trial compared SZC efficacy and safety; (78)	SZC 10 g TID for 24–72 h until normokalemia followed by once daily SZC 5 g for 12 months	Hyperkalemia (potassium >5.1 mmol/L) and CKD (4 and 5) vs. those CKD (1–3) >12 months *N* = 751	Correction Phase: 82% of patients achieved normokalemia in both eGFR within 24 h, 100 and 95% with eGFR <30 and ≥30 mL/min, respectively, within 72 h
PRIORITIZE-HF: Phase 2	SZC compared to placebo.	Patients with HF taking RAASi *N* = 182	Ongoing (NCT03532009).

*BB, Beta blockers; CKD, Chronic Kidney Disease; DM, Diabetes mellitus; HF, Heart Failure; HT, Hypertension; RAASi, Renin Angiotensin Aldosterone System Inhibitors*.

Under *in vitro* conditions mimicking the pH and potassium content of the colon, Patiromer binds 8.5–8.8 mmol of potassium per gram of polymer (80). In healthy volunteers, Patiromer administered for 8 days three times a day, caused a dose-dependent increase in fecal potassium excretion, with a corresponding dose-dependent reduction in urinary excretion (81).

The PEARL HF study explored the safety/efficacy profile of Patiromer in a large population of patients with HF and either a history of hyperkalemia resulting in the discontinuation of RAASi or MRA, or CKD treated with one or more HF therapies. Patiromer was associated with a significantly lower serum potassium levels, a lower incidence of hyperkalemia, and a higher proportion of patients on spironolactone after 4 weeks (65) (Table 3). Patiromer was also tested for 4 weeks in hyperkalemic CKD stage 3–4 patients undergoing stable treatment with one or more RAASi in the OPAL-HK study (66), showing a stable potassium lowering levels in two phases, regardless of age, gender, baseline potassium levels, diabetes, HF and maximal/not maximal RAASi dosage (82). An interesting finding of OPAL-HK study was the reduction of aldosterone levels independent of plasma renin activity, hyperkalemia or use of RAASi, followed by a reduction in blood pressure and albuminuria. This finding comes up with the idea that Patiromer may improve cardiovascular risk beyond the reduction in potassium levels (83).

The recent AMBER study, evaluated the use of Patiromer in patients with RHT and CKD. Patiromer allowed to 86% of the patients to remain on spironolactone with less hyperkalemia after 12 weeks, what has a critical relevance in the treatment of RHT (34). Finally, the ongoing DIAMOND study, that will end in 2022, will determine whether Patiromer treatment of HF subjects with hyperkalemia while receiving RAASi allows to continue RAASi considering not only safety or efficacy endpoints but primary “hard” endpoints (time to the first occurrence of cardiovascular death or hospitalization) (84).

Based on the above-mentioned large trials, Patiromer is recommended at a starting dosage of 8.4 g once daily, administered orally, which can be increased by 8.4-g increments per week, titrated up to a maximum of 25.2 g once daily (79).

Sodium Zirconium Cyclosilicate (SZC) is the most recently approved potassium binding agent with efficacy and safety established in phase 2 and 3 clinical trials of patients with hyperkalemia and CKD, HF, and/or diabetes or those receiving RAASi (Table 4). SZC is a non-absorbed, insoluble, inorganic crystal that selectively entraps potassium in the GI tract in exchange for sodium and hydrogen. Because of its high selectivity for potassium, SZC may bind it throughout the entire GI tract, and may produce a rapid potassium lowering effect. It has been estimated that 1 g of SZC binds about 3 mmol of potassium, and its activity begins within 1 h after taking (85).

SZC has been evaluated in several randomized trials and open-label long-term observational studies (Table 4). In the HARMONIZE study, hyperkalemic patients with CKD, HF, or diabetes received SZC for 48 h and showed a significant reduction in potassium levels. Ninety-eight percentage of patients achieved normokalemia after a week. Then, after achieving normokalemia, SZC reduced potassium during days 8 through 29 in a dose-dependent way (73, 78). The ENERGIZE trial tested SZC in patients admitted at the Emergency Department (ED) with acute hyperkalemia. SZC was administered up to three times during 10-h, in association with insulin and glucose compared to placebo. Reductions in potassium levels at 1 h with SZC or the placebo were similar, probably due to the predominant potassium-lowering effect of the concomitant insulin and glucose treatment, but a greater reduction in mean potassium was observed in the SZC compared with the placebo at 2 h suggesting the beneficial role of SZC in the emergency treatment of hyperkalemia (72).

The ZS-005 trial tested the long-term efficacy and safety of SZC in 751 outpatients with hyperkalemia. Ninety-nine percentage of the patients achieved potassium of 3.5–5.5 mmol/L in the correction phase, while more than 90% of the patients achieved a normal potassium level after 12 months of treatment (77).

In hyperkalemic patients undergoing hemodialysis, once-daily SZC on non-dialysis days effectively maintained pre-dialysis serum potassium levels on three out of four dialysis treatments, after long interdialytic period without rescue treatment over 8 weeks in the DIALIZE study. Interestingly, adverse effects, including interdialytic weight gain, were similar between the two groups (71). The ongoing PRIORITIZE-HF trial (estimated to end in 2020) (NCT03532009) will evaluate the effect of SZC compared to placebo in patients with HF taking RAASi.

SZC is dosed 10 mg TID in the acute phase and dropped to 5–10 mg daily thereafter. Given that SZC is insoluble, is not systemically absorbed and does not expand on contact with water, it is very well-tolerated.

### Safety and Tolerability of News Potassium Binders

Patiromer and SZC are generally well-tolerated. Overall, Patiromer related adverse events occurred in ~20% of the patients enrolled the major trials (54). These events include electrolyte disorders, such as hypomagnesemia and hypokalemia, and mild gastrointestinal symptoms, such as constipation (8%), diarrhea (5%), nausea and flatulence (65). Monitoring serum magnesium is recommended, considering supplementations for patients who develop hypomagnesemia while on patiromer (86). *In vitro* studies indicated that Patiromer may interact with some medications like ciprofloxacin, levothyroxine and metformin (87). Therefore, the administration of other oral medications at least 3 h before or 3 h after Patiromer is recommended.

SZC has not been associated with any serious adverse effects in RCTs. The most common were hypokalemia (5.8% of patients enrolled in different studies) and a dose-dependent increase in edema (88) cause because its sodium content. Monitoring signs of edema, especially in patients at risk of fluid overload, such CKD and CHF patients is recommended, and adjusting dietary salt intake and the dose of diuretics is probably required (89). In phase 2 and 3 trials, the incidence of gastrointestinal adverse events (nausea, constipation, vomiting or diarrhea) was similar between the treated group and the placebo group. However, as for patiromer, SZC should also not be used in patients with severe constipation, bowel obstructions or impaction, including abnormal postoperative bowel motility disorders. Because SZC may affect absorption of other oral medications with pH-dependent solubility due to a transient increase in gastric pH, SZC administration should be separated from these medications by at least 2 h (85),

## Summary

Hyperkalemia is a common complication in patients with comorbidities (CKD, diabetes, HF) and among those taking certain critical medications (RAASi and MRAs). The frequency and severity of hyperkalemia increases during CKD progression, and is associated with higher mortality. As RAAS inhibition augments the risk of hyperkalemia, improvement in potassium control could allow enhancing RAAS inhibitor use in patients with an evidence-based indication. Cation exchange resins used to treat hyperkalemia are unsuitable for long-term use owing to gastrointestinal side effects. Newer potassium-lowering therapies (Patiromer and SZC) can effectively and safely correct hyperkalemia and maintain normokalemia in patients with co-morbidities receiving RAASi therapy. The long-term efficacy and safety of newer potassium-binders remains to be ascertained. However, their use for cardiovascular and renal risk reduction in combination with RAASi therapy holds promise for renal and cardiovascular protection in non ND-CKD patients. Nowadays this represents one of the most important news released to the renal community.

## Author Contributions

EM, PC, and JM contributed equally in the writing and reviewing the paper. All authors contributed to the article and approved the submitted version.

## Conflict of Interest

EM and JM report serving as consultants participating in advisory boards for Vifor Pharma Group Company. The remaining author declares that the research was conducted in the absence of any commercial or financial relationships that could be construed as a potential conflict of interest.
